# Impact of Preoperative Calcium and Magnesium Supplementation on Quality of Life and Hypocalcemia Post‐Thyroidectomy

**DOI:** 10.1002/edm2.70129

**Published:** 2025-11-30

**Authors:** Navid Tabriz, Dennis Fried, Verena Uslar, Dirk Weyhe

**Affiliations:** ^1^ Pius‐Hospital Oldenburg, University Hospital for Visceral Surgery Carl von Ossietzky University Oldenburg Oldenburg Germany

**Keywords:** calcium, magnesium, quality of life, thyroidectomy

## Abstract

**Objective:**

Postoperative hypocalcemia and hypoparathyroidism are common complications after thyroidectomy, often impairing quality of life (QoL). This study investigates the impact of preoperative calcium and magnesium supplementation on postoperative QoL and hypocalcemia in patients undergoing total thyroidectomy for symptomatic nodular goitre or Graves' disease.

**Methods:**

A total of 62 patients undergoing thyroidectomy for benign thyroid diseases were randomised into two groups. The intervention group (IG, *n* = 31) received 500 mg calcium carbonate thrice daily and 300 mg magnesium carbonate once daily for 2 weeks preoperatively, while the control group (CG, *n* = 31) received no supplementation. Laboratory parameters (Ca, Mg, PTH, 25‐OH‐Vitamin D) were measured at study enrolment (T_1_), preoperatively (T_2_), immediately postoperatively (T_3_) and 6 weeks post‐discharge (T_4_). QoL was assessed using EQ‐5D and ThyPro39de questionnaires.

**Results:**

QoL significantly improved postoperatively in both groups. Patients with Graves' disease in the IG reported earlier QoL improvements immediately post‐surgery (T_3_). Postoperative hypocalcemia occurred in 19.4% of IG patients and 25% of CG patients, with hypoparathyroidism in 16% and 23%, respectively. The IG demonstrated higher postoperative calcium levels and fewer hypocalcemia symptoms, especially in Graves' disease patients (not significant). Vitamin D deficiency was prevalent (66.7%) but showed no correlation with hypocalcemia.

**Discussion:**

Preoperative calcium and magnesium supplementation might have positive effects on postoperative QoL, especially in Graves' disease patients, and may reduce hypocalcemia symptoms. This simple, inexpensive and low‐risk intervention may be beneficial in the preoperative setting prior to thyroidectomy. Although the observed effect did not reach statistical significance, it could still be of clinical relevance. The additional benefit of preoperative magnesium supplementation seems to be of minor significance, while the effect of pre‐existing vitamin D deficiency remains uncertain.

## Graphical abstract

Postoperative QoL improved in both groups, with earlier gains in Graves' disease patients receiving supplementation. Hypocalcemia and hypoparathyroidism rates were lower in the intervention group, which also showed higher calcium levels and fewer symptoms. Vitamin D deficiency was common but unrelated to hypocalcemia.

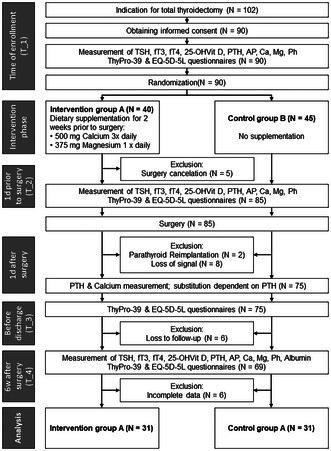

**Abbreviations**
ANOVA: analysis of variance
Ca: calcium
CG: control group
EQ5D: European Quality of Life 5 Dimensions
GD: Graves' disease
IG: intervention group
Mg: magnesium
NG: nodular goitre
PTH: parathyroid hormone
T_1: study inclusion, usually 2 weeks before surgery
T_2: usually 1 day preoperative
T_3: 2 days postoperative
T_4: 6 weeks postoperative
*ThyPRO*
: thyroid‐related patient‐reported outcome
VAS: visual analogue scale

## 1 Introduction

Postoperative hypocalcemia mostly due to hypoparathyroidism is the most common complication after thyroidectomy. Its incidence ranges from 0.3% to 49% [1, 2]. The supplementation of activated vitamin D and calcium is provided to treat postoperative hypoparathyroidism, based on the levels of postoperative PTH [3]. PTH levels of < 15 pg/mL, measured at least 20 min after thyroidectomy, can assist in predicting potentially clinically significant hypoparathyroidism and early substitution therapy [3, 4]. Conversely, a PTH threshold of > 19.5 pg/mL in the immediate postoperative phase can serve as a marker for safe early discharge [5]. Despite this clinically common treatment protocol, there is still no universally accepted recommendation in the literature for the management of postoperative hypoparathyroidism.

The symptoms of postoperative hypocalcemia range from mild paresthesia and muscle cramps to laryngospasms and life‐threatening cardiac arrhythmias. These symptoms can lead to a prolonged hospital stay and significantly reduce the patient's quality of life (QoL) [6, 7]. The development of postoperative hypoparathyroidism is multifactorial. Graves' disease is associated with nearly double the rate of postoperative hypoparathyroidism compared to nodular goitre (30% vs. 15% in Germany) [8]. However, the surgeon's expertise and the extent of the surgery also play a crucial role as risk factors [9, 10]. The impact of vitamin D levels on the development of postoperative hypocalcemia is a subject of controversy in the literature [11, 12]. Thyroidectomy can induce significant hypomagnesemia, which may correlate with the onset and duration of hypocalcemia [13, 14].

This raises the question of whether, in addition to optimised surgical techniques, preoperative measures can be implemented to positively influence the occurrence and course of postoperative hypocalcemia, thereby improving the quality of life for patients.

The primary endpoint of this study was to investigate whether preoperative supplementation with calcium and magnesium has a positive impact on quality of life, as measured by the ThyPRO composite score over time, in patients undergoing thyroidectomy for Graves' disease (GD) and nodular goitre (NG). Secondary endpoints included (i) an additional assessment of quality of life using the EQ‐5D questionnaire, (ii) evaluation of the potential effect of supplementation on the incidence of postoperative hypocalcemia, and (iii) investigation of the possible impact of vitamin D on postoperative hypocalcemia.

The long‐term goal was to demonstrate a positive outcome from preoperative supplementation and potentially provide a recommendation for future preoperative management prior to thyroidectomy.

## 2 Methods

### 2.1 Study Desgin

A monocentric prospective randomised controlled intervention study with a postoperative follow‐up of 6 weeks was conducted. Informed written consent was obtained from all patients. The study was registered with the German Clinical Trials Registry (DRKS; Study ID: DRKS00017195; Date of registration: 22 May 2019) and was initiated after study registration. It was approved by the ethics committee of the Carl von Ossietzky University Oldenburg (vote number: 2017‐105), and it was performed in accordance with relevant guidelines and regulations, especially the CONSORT statement. A block randomization with a block length of 6 was applied. The randomization script was written in Matlab by a researcher, who was not involved in informing the patients or collecting the study data. The researcher prepared numbered envelopes in which slips of paper with the allocation specified by the results of the randomization script were inserted. The envelopes were opened in the correct order by the informing physician in the presence of the patient after they had agreed to participate in the study. For further details we also refer to our publication of the study protocol [15].

**FIGURE 1:**
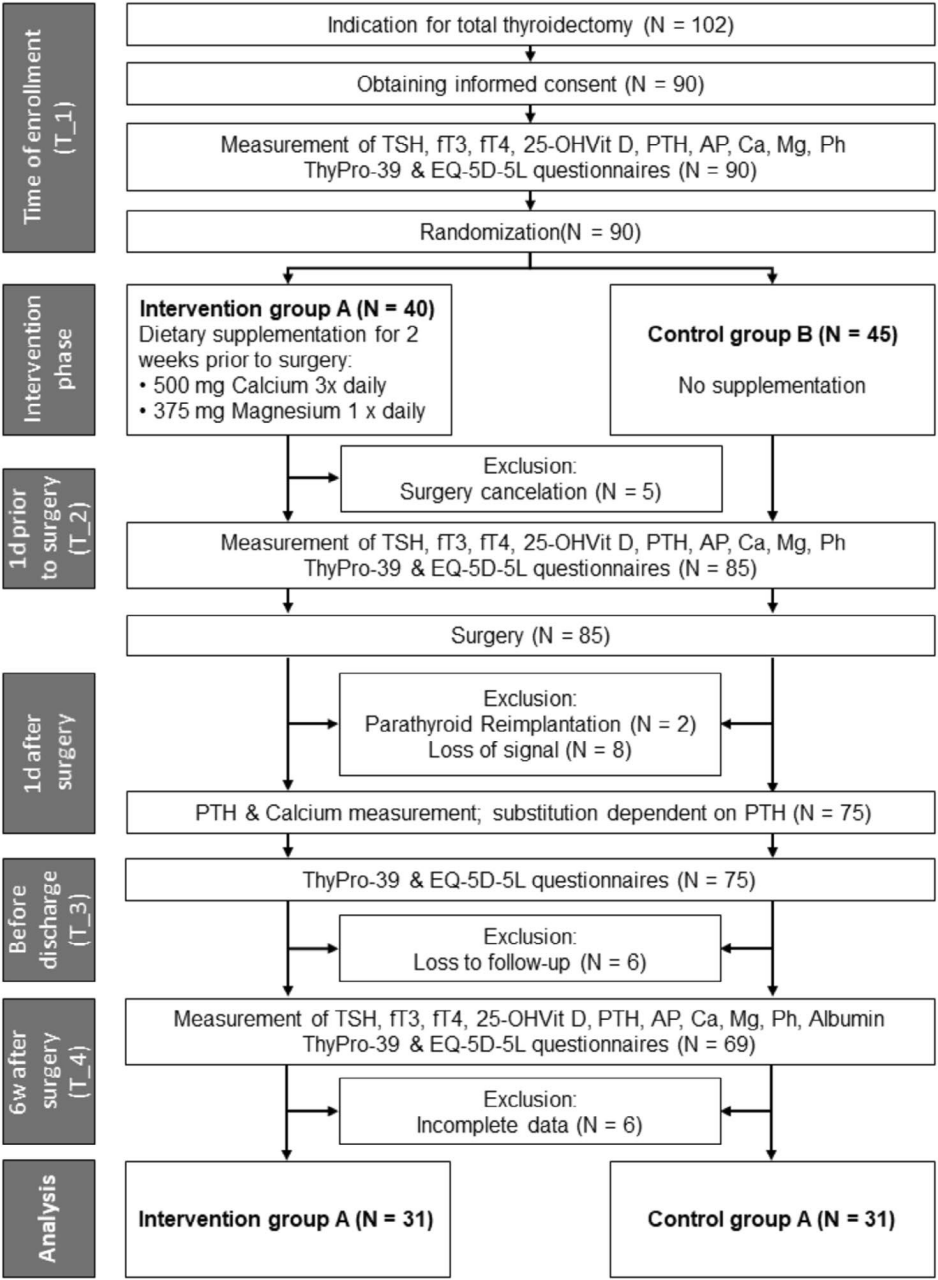
Study flow chart.

In summary, after the indication for thyroidectomy was established due to symptomatic or suspected thyroid nodules/goitre (NG), or Graves' disease (GD), patients were randomised into an intervention group (IG), which received supplementation with calcium 3 × 500 mg/day (Calcium Sandoz 500 mg, Hexal, equivalent to 1250 mg calcium carbonate) and magnesium 1 × 375 mg/day (Magnetrans 375 mg, StadaVital, nearly equivalent to 625 mg magnesium oxide) for 2 weeks prior to surgery, and a control group (CG) without nutritional supplements (T_1) (Figure 1). Participants were explicitly instructed not to take any additional supplements or vitamin products.

Both groups were also asked to complete the ThyPRO‐39de [16] and EQ‐5D‐5L (EQ‐5D) [17] QoL questionnaires, and a laboratory test was performed. One week after starting the supplements, participants were contacted by phone to evaluate their well‐being and inquire about any side effects. One day before the planned surgery (T_2), both groups underwent another QoL assessment and laboratory testing and it was reconfirmed verbally that the participants had not taken any additional supplements or vitamins. Thyroidectomy was performed under intermittent intraoperative neuromonitoring of the recurrent laryngeal nerves. Intraoperatively, the macroscopic view of the parathyroid glands and description of structure and blood flow were required. If the lower parathyroid glands were not found in loco typico, an explicit representation was not necessary. In the case of an accidentally removed parathyroid gland or insufficient blood supply, the gland was removed, cut into small cubes and autologous reimplanted in a muscle pocket of the sternocleidomastoid muscle. The affected patient was then excluded from the study. 6–24 h postoperatively, parathyroid hormone (PTH) and calcium levels were measured. Based on these results, it was decided whether calcium ± vitamin D supplementation was necessary. Additionally, the patients were evaluated for the presence of hypocalcemia‐related symptoms, such as paraesthesia of the fingertips or perioral region (Table 1). QoL was reassessed before hospital discharge (T_3). Six weeks after surgery (T_4), another laboratory test and QoL assessment were conducted.

**TABLE 1:** Postoperative procedure dependent on PTH levels (6–24 h postoperatively).

	PTH < 15 pg/mL	PTH 15–30 pg/mL	PTH 30–65 pg/mL	PTH > 65 pg/mL
Alphacalcidol	0.5 μg 2 × d	—	—	—
Calcium carbonate	2 × 1 g	2 × 1 g	As required 1 g	As required 1 g
Magnesium oxide	2 × 375 mg	375 mg	—	—

### 2.2 Statistics

Data collection was performed using Microsoft Excel 2019. Statistical analyses and graphical representations were conducted using the statistical software SPSS Version 30 and JASP Version 0.19.3, as well as an online tool for calculation of the 95% confidence interval of the effect size ($\eta_{p}^{2}$) and figures were created using Origin version 2021b [18]. The EQ‐5D and ThyPro39de questionnaires were evaluated according to existing recommendations. All analyses were performed according to intention‐to‐treat. Patient characteristics, QoL questionnaire scores and laboratory results were analysed descriptively, separated by intervention and follow‐up period. For all categorical variables, counts and percentage frequencies were used. For continuous variables, means and standard deviations were calculated. Patient age was reported alongside the mean with minimum and maximum values.

Inferential statistics were conducted using repeated measures ANOVA between the factors (i.e., supplementation or not) for the primary endpoint, QoL over time, as measured by the ThyPro39de. A repeated measures analysis was applied to the following outcome variables: laboratory values (Ca, Mg, PTH, 25‐OH vitamin D), EQ‐5D questionnaire and ThyPro39de questionnaire. The repeated measures analysis differentiated between intervention group A and control group B, with further differentiation into subgroups based on the underlying condition (Graves' disease or multinodular goitre). Additionally, box plots were generated for the aforementioned variables. All *p*‐values of the post hoc pairwise comparison tests were corrected using Holm's correction.

Subdomains of the ThyPRO39de were analysed descriptively and means of the sub domains at T_1 and T_4 were plotted in radar plots for all patients, and for patients with GD and NG separately.

The Chi‐squared test or Fisher's exact test was used to determine differences between the groups in the development of postoperative clinical hypocalcemia. A *p*‐value < 0.05 was considered statistically significant for all tests.

The sample size of approximately 90 individuals was calculated for the validation of the ThyPro39de questionnaire, based on standard metrics used for questionnaire validation. Due to the exploratory nature of the study and the lack of comparable data, no sample size calculation could be performed initially for the measurement of quality of life. Therefore, power analysis was conducted post hoc via G*Power [19]. For QoL assessment measured by the ThyPro39de, a total of *n* = 76 participants would be required to detect a significant interaction effect in a repeated measures ANOVA with two groups (given by diagnosis) and four measurements (four visits) and an *α* = 0.05, an effect size of $\eta_{p}^{2}$ = 0.047 for the interaction diagnosis*time for ThyPro39de (equivalent to an f of approximately 0.222) and a power of 0.8.

## 3 Results

### 3.1 Patient Characteristics

Between March 2019 and May 2022, a total of *n* = 90 patients were enrolled in the study (see Figure 1). Five patients had to cancel the operation at short notice. Therefore *n* = 85 patients were randomised, *n* = 40 in the IC group A and *n* = 45 in the CG group B. Out of these, *n* = 23 patients had to be excluded: *n* = 2 due to parathyroid gland reimplantation, *n* = 8 due to intraoperative loss of neuromonitoring signal and therefore only hemithyroidectomy, and *n* = 13 due to loss of follow‐up and incomplete data. This resulted in an evaluable total collective of *n* = 62 patients, which were evenly divided into the two groups of *n* = 31 patients each. No participants in the IC group experienced adverse effects related to the intake of the supplements. In cases of immediate postoperative hypocalcemia or hypoparathyroidism, the prescribed regimen (Table 1) was administered in all instances in accordance with our institution's standard operating procedure. The group composition is shown in Table 2.

**TABLE 2:** Biometric data and diagnoses of the study patients.

	Intervention group A	Control group B	Total collective
Male (*n*/%)	10/32	3/10	13/21
Female (*n*/%)	21/68	28/90	49/79
Graves' disease (*n*/%)	16/52	16/52	32/52
Nodular goitre (*n*/%)	15/48	15/48	30/48
Mean age (min/max)	47 (20–70)	49 (20–72)	48 (20–72)
Mean BMI (min/max)	32 (22–45)	28 (20–48)	30 (20–48)

### 3.2 Quality of Life

Quality of life was assessed at four different time points (T_1: 2 weeks preoperatively before the start of supplementation, T_2: 1 day preoperatively, T_3: before inpatient discharge, usually the 3rd postoperative day, T_4: 6 weeks postoperatively) using two questionnaires.

#### 3.2.1 ThyPRO‐39de Questionnaire

**FIGURE 2:**
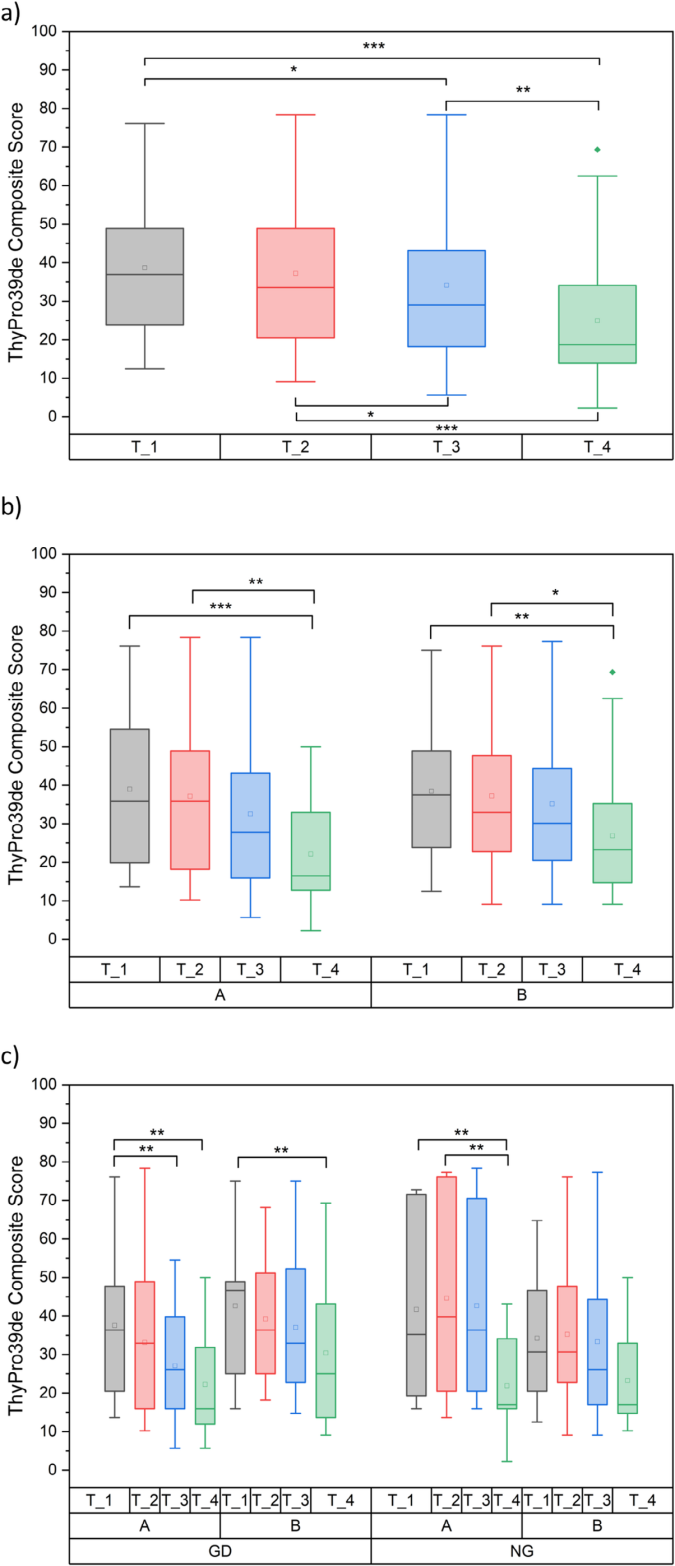
ThyPRO‐39 Composite Score analysis (0 = best QoL, 100 = worst QoL), (a) total collective, (b) divided into IG A and CG B, (c) GD/NG divided into IG A and CG B. The boxes contain the 25% and the 75% quartile as well as the median, black dots depict the mean, and whiskers range from the 5% percentile to the 95% percentile. *, **, *** indicate significant differences between the respective groups.

In the overall collective, a steady significant improvement in QoL was recorded postoperatively independent of supplementation (*p* < 0.001; effect size of $\eta_{p}^{2}$ = 0.305), and there was a significant difference between each visit (all *p* ≤ 0.025; Cohen's > 0.247 for all significant comparisons), except for T_1 versus T_2 (Figure 2a; *p* = 0.294; Cohen's *d* = 0.077). When calculated separately for Group A (intervention) and Group B (control), both groups had identical baseline values at the start of the study (T_1) (Group A (mean and SD): 36.7 ± 20.3; Group B: 36.7 ± 17.3; see also Figure 2b). However, by the time of the final measurement (T_4), the composite score improved in Group A to 20.7 ± 12.8 (*p* < 0.001; Cohen's *d* = 0.932) and in Group B to 26.0 ± 15.4 (*p* = 0.002; Cohen's *d* = 0.620). Group A and Group B also showed a significant difference for T_2 versus T_4 (*p* = 0.005 and 0.036, and Cohen's *d* = 0.847 and 0.551, respectively) (Figure 2b).

There was no significant interaction effect of time and diagnosis (*p* = 0.066; $\eta_{p}^{2}$ = 0.047). However, there seems to be a tendency towards GD patients reaching better QoL faster than NG patients (see Figure 2c).

When differentiated between time, intervention versus control, and GD versus NG, all groups showed a significant improvement from T_1 to T_4 (all *p*‐values ≤ 0.002), except for the NG control group. In addition, for the intervention groups there was a significant difference between T_1 and T_3 (*p* = 0.001) in the GD group, and between T_2 and T_4 in the NG group (*p* = 0.002). At T_4, both intervention groups showed better scores than their respective control groups (mean and SD: GD intervention 22.4 ± 14.7 vs. GD control 28.2 ± 18.2; NG intervention 20.3 ± 13.3 vs. NG control 23.3 ± 11.3) (Figure 2c).

**FIGURE 3:**
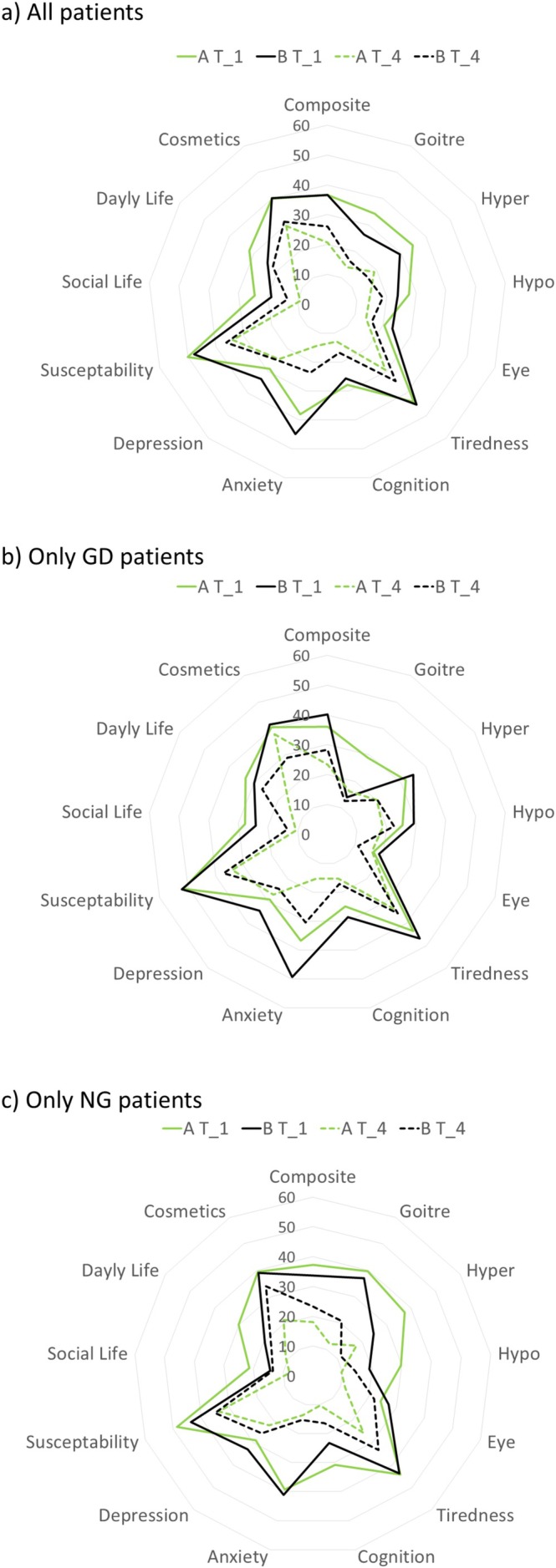
Radar plots for the subdomains of the ThyPRO (mean values from T_1 to T_4), (a) total collective Group A (IC) versus Group B (CG); (b) GD, IC versus CG; (c) NG, IC versus CG; lower values better QoL.

The ThyPRO questionnaire is made up of 12 symptom categories. However, the symptom‐specific categories (goitre symptoms, hyper‐ and hypothyroidism symptoms, eye symptoms) are not subsumed in the composite score. For this reason, all categories are demonstrated as radar plots, comparing T_1 and T_4 between Group A and Group B (Figure 3).

With regard to the subdomains except impaired social life and daily life, a marked improvement in QoL 6 weeks after surgery (T_4) was demonstrated both in the intervention group and the control group. However, in all domains except hyperthyroidism symptoms patients in Group A showed better QoL than patients in Group B in T_4, although in most domains except anxiety and depression Group A patients showed equal or even worse QoL than Group B patients at T_1 (Figure 3a).

This effect seems mostly due to the NG patients, since the pattern (i.e., Group A's subdomain scores improving more than Group B's) is even more pronounced (compare Figure 3a,c). As for the GD patients, both groups' QoL subdomain scores increase between T_1 and T_4, with Group A reaching markedly better scores only in the anxiety subdomain as well as the daily life and the composite score when compared to Group B (Figure 3b). In all other subdomains except cosmetics Group A and Group B were comparable. Notably, the cosmetics subdomain score did not change for Group A between T_1 and T_4.

#### 3.2.2 EQ‐5D Questionnaire

The EQ‐5D questionnaire is a disease‐unspecific general quality of life questionnaire, in which five domains (mobility, independent care, everyday activities, pain/physical discomfort and anxiety/depression) are subsumed.

**FIGURE 4:**
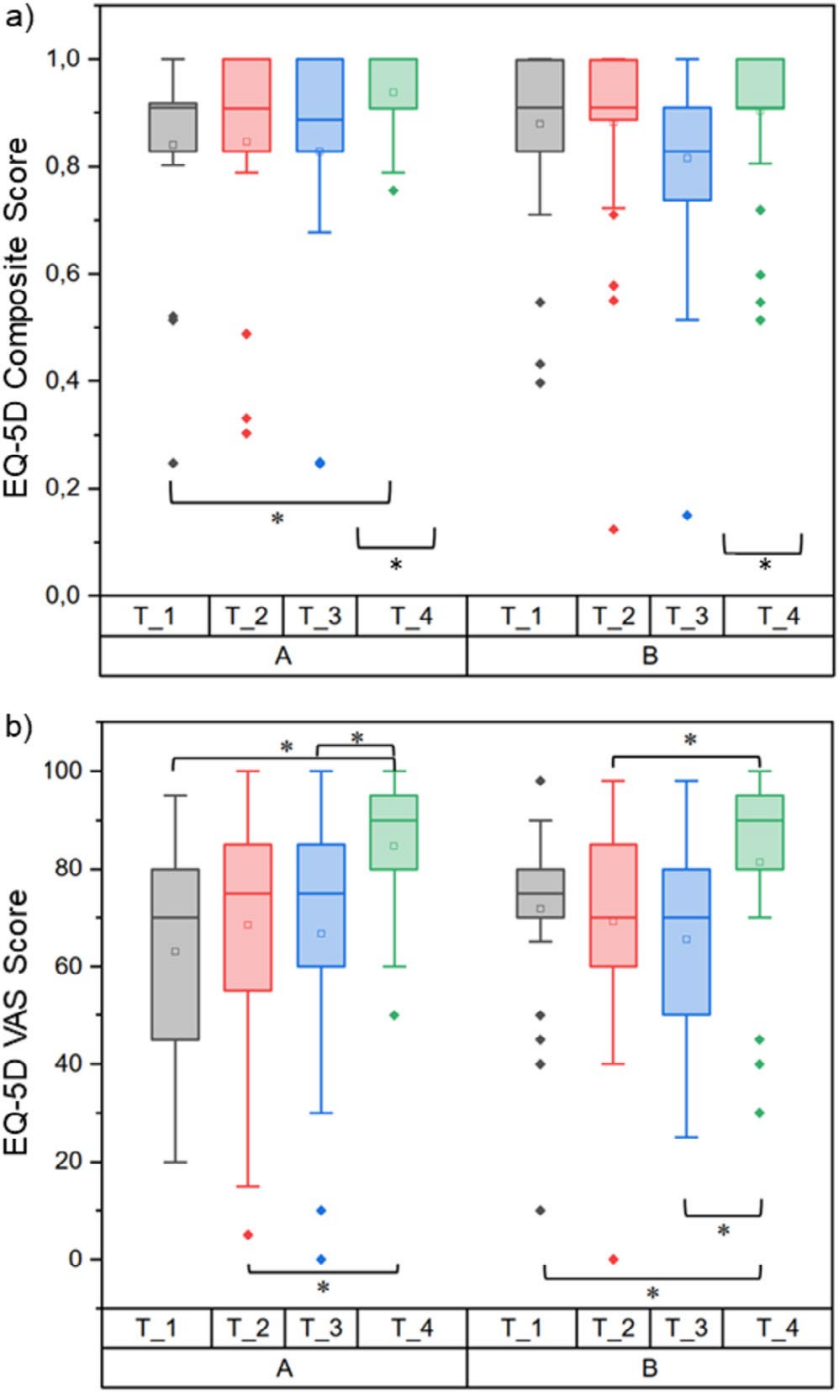
Quality of life EQ‐5D questionnaire in group comparison; (a) EQ‐5D composite score, (b) EQ‐5D VAS (Score 0 = worst quality of life, score 100 = best quality of life). The boxes contain the 25% and the 75% quartile as well as the median, black dots depict the mean, and whiskers range from the 5% percentile to the 95% percentile. *significant differences between the groups.

In the IG group (*p* < 0.001 [95% CI: 12.22–30.39]) as well as in the CG group (*p* = 0.005; 95% CI: 2.25–17.03), a significant improvement in QoL preoperatively to 6 weeks postoperatively was recorded in the composite score, and the VAS. Both groups also showed a significant improvement from the time of discharge to 6 weeks after the operation (Figure 4a,b).

There was no difference in either part of this questionnaire between the groups, even depending on the diagnosis of GD versus NG.

### 3.3 Laboratory Parameters

#### 3.3.1 Calcium and Parathyroid Hormone (PTH)

**FIGURE 5:**
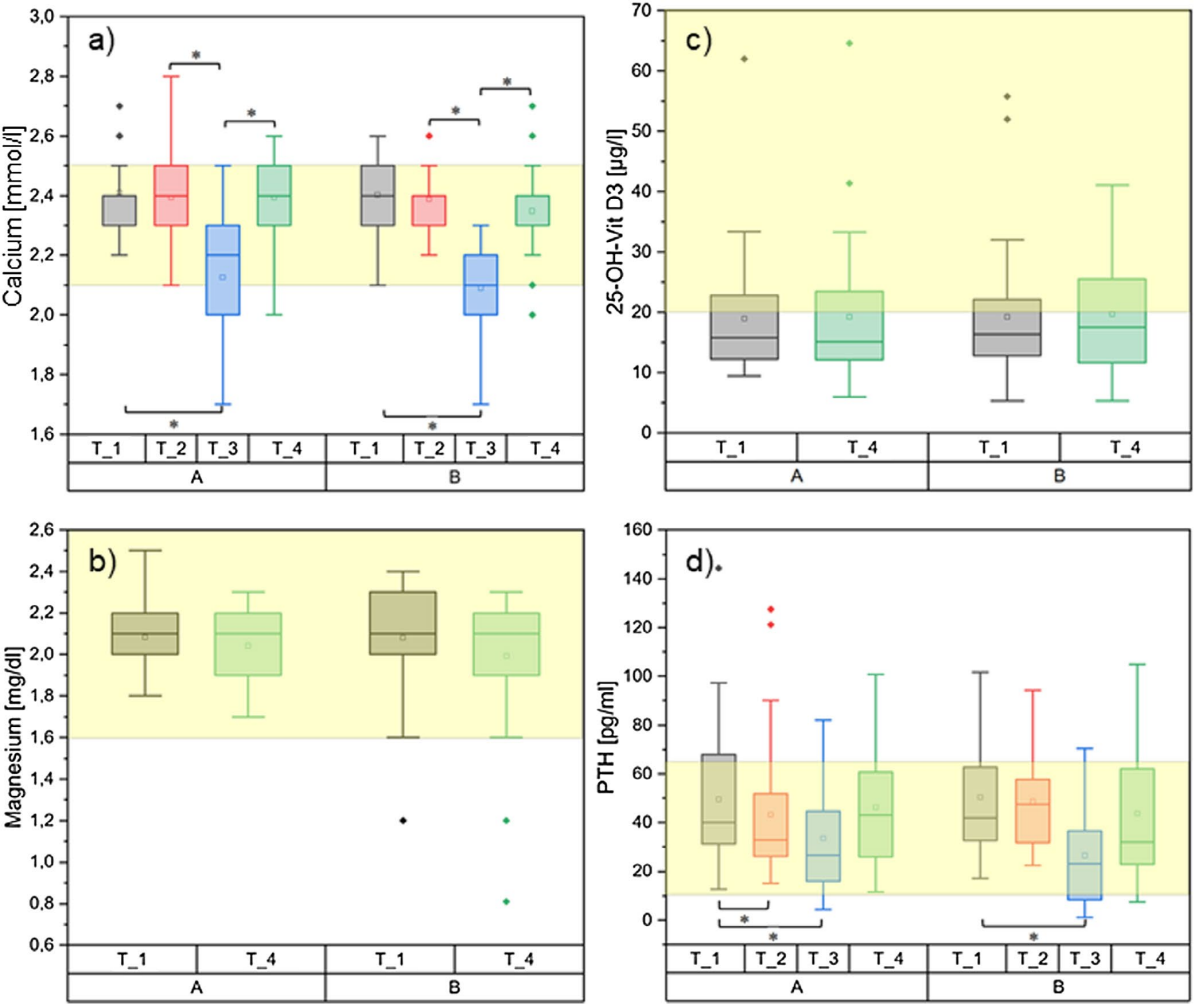
Course of the laboratory parameters (a) Serum total calcium (norm 2.2–2.6 mmol/L), (b) Magnesium (norm 0.7–1.1 mmol/L), (c) 25‐OH‐vitamin D, (d) Parathyroid hormone (norm 15‐65 pg/mL), A = intervention group, B = control group, yellow marked area = normal value. *significant differences between the groups.

Both groups showed a significant drop in calcium levels immediately postoperatively with normalisation after 6 weeks (see also Figure 5a). A significant difference between the groups and depending on the diagnosis of GD and NG could not be demonstrated. In total, 14 patients developed hypocalcemia immediately postoperatively. Of these, sixbelonged to the intervention group (two with Graves' disease and four with nodular goitre) and eight to the control group (five with Graves' disease and three with nodular goitre). Among these patients with hypocalcemia, those with Graves' disease all also had hypoparathyroidism (Table 3). At the 6‐week follow‐up, all patients were asymptomatic with normal PTH levels, except for one asymptomatic patient with a slightly below‐reference PTH level of 14.7 ng/mL (Figure 5d).

**TABLE 3:** Rate of subjects with hypocalcemia and hypoparathyroidism at timepoint T_3 (postoperative), stratified by intervention group, diagnosis and presence of symptoms, IG (Intervention Group), CG (Control Group).

	IG		CG	
	Graves' disease (*n* = 16)	Nodular goitre (*n* = 15)	Graves' disease (*n* = 16)	Nodular goitre (*n* = 15)
Postoperative hypocalcemia *n* (%)	2 (12.5)	4 (26.7)	5 (31.3)	3 (20)
Postoperative hypoparathyroidism *n* (%)	2 (12.5)	3 (20)	5 (31.3)	2 (13.3)
Symptomatic (paraesthesia) *n* (%)	2 (12.5)	2 (13.3)	4 (25)	2 (13.3)
Asymptomatic *n* (%)	0	1 (6.7)	1 (6.3)	0

In the IC group, calcium supplementation resulted in a significant drop in PTH 2 weeks after the start of calcium intake (*p* = 0.042; 95% CI: 0.15–12.79). However, the value remained within the reference range and served as evidence for physiological regulation and as proof of supplement intake. Immediately postoperatively, there was a significant drop in the PTH value in both groups (IG 32.8 pg/mL [95% CI: 24.57–41.03] *p* = 0.028 and CG 26.66 pg/mL [95% CI: 18.88–32.43] *p* < 0.001), which normalised again after 6 weeks.

#### 3.3.2 Magnesium

Magnesium levels were in the normal range over the supplementation period with no difference between the groups (Figure 5b).

#### 3.3.3 Vitamin D

The measurement of 25‐OH‐Vitamin D3 was conducted at study inclusion (T_1) and 6 weeks post‐hospitalisation (T_4). 66.7% of all patients had a vitamin D deficiency (Figure 5c). Of the 40 patients with Vitamin D deficiency, 20 were randomly assigned to Intervention Group A and 20 to Control Group B. In the IG A, the mean value at study inclusion (T_1) was 18.52 μg/L (95% CI: 14.4–22.63), and at the post‐hospitalisation measurement, it was 18.97 μg/L (95% CI: 14.95–23). In the CG B, the mean value at T_1 was 19.1 μg/L (95% CI: 15.12–23.08), and 19.6 μg/L (95% CI: 15.71–23.5) by the end of the study. No significant differences were found between the IG and CG groups or between the GD and NG groups.

## 4 Discussion

Improving quality of life is one of the main goals in thyroid surgery, especially for diseases such as symptomatic nodular goitre or Graves' disease. Therefore, perioperative morbidity should be kept as low as possible [20]. Postoperative hypocalcemia is the most common complication of thyroidectomy and can deteriorate patients' quality of life. Additionally, hypomagnesemia can significantly negatively impact the onset, duration and modulation of hypocalcemia [14].

Most of the present studies have investigated whether short‐term postoperative calcium, magnesium and/or vitamin D supplementation can reduce the severity of postoperative hypocalcemia following thyroidectomy. Despite several positive findings, no clear recommendation has been established for the prevention or early therapy of postoperative hypocalcemia. However, the influence of prophylactic calcium and magnesium supplementation on patients' quality of life with an appropriate QoL questionnaire has not been investigated yet. For an accurate assessment of health‐related QoL, the combination of generic and disease‐specific questionnaires is recommended [16]. For this reason, the combination of the ThyPRO‐39de and EQ‐5D questionnaires was used in this study. In general, two prospective studies with a 12‐month follow‐up demonstrated that the quality of life of patients following thyroid surgery, as measured by the EQ‐5D questionnaire, tends to improve [21, 22]. A meta‐analysis from 2022, which included a total of six studies with a cumulative 496 patients, demonstrated that an improvement in quality of life according to ThyPRO criteria was observed in all domains following surgical treatment of the thyroid for benign indications [23]. We can clearly demonstrate that the postoperative quality of life steadily improves after surgery and already shows a significant improvement after 6 weeks, independent of supplementation using both the ThyPRO‐39de and the EQ‐5D questionnaire.

The preoperative supplementation did not affect the QoL significantly but supplemented patients showed overall lower, and thus better, quality‐of‐life scores in the ThyPRO questionnaire, particularly in the GD group, where the mean composite score of patients in the intervention group is about 6 points lower, that is better, than in the comparison group and thus at the lower end of the minimal important change (MIC) given for the ThyPRO in its original language (MIC: 6.3–14.3 for groups and 8.0–21.1 for individuals) [24]. Moreover, patients with supplemented Graves' disease showed a significant improvement in quality of life immediately postoperatively compared to all other groups. Notably, the categories of depression, eye symptoms, fatigue and emotional vulnerability improved already during the postoperative phase. This postoperative effect was not observed in the other subgroup. Patients with Graves' disease are more susceptible to anxiety and depressive symptoms compared to those with nodular goitre. Magnesium also plays a modulatory role in the brain's signalling cascades and can be used as supportive therapy in antidepressant treatment [25, 26]. Therefore, it can be assumed that supplementation therapy with calcium and magnesium, particularly in patients with Graves' disease, may lead to an improvement in both psycho‐vegetative and somatic symptoms. Their preoperative supplementation might be therefore important for daily clinical practice although without statistical significance in this study.

When interpreting the EQ‐5D questionnaire, it should be considered that patients already started with high baseline values in the composite score, which may limit interpretation due to ceiling effects. Overall, the additional use of a generic questionnaire alongside ThyPRO in future quality‐of‐life studies appears to be dispensable, as it does not provide further information gain. Our working group has previously issued recommendations on the use of quality‐of‐life questionnaires in thyroid diseases in a systematic review [27].

The secondary endpoint of the study was to examine the effect of the preoperative supplementation therapy on the course of postoperative hypocalcemia. The extent of surgical radicality, Graves' disease, accidental removal of parathyroid glands and their autotransplantation, as well as surgical expertise, are risk factors for postoperative hypocalcemia [28, 29, 30]. For this reason, patients with autotransplanted parathyroid glands were excluded from the analysis. Approximately 24% of all patients after thyroidectomy develop hypocalcemia on the first postoperative day [9]. In clinical practice, calcium with or without activated vitamin D is usually initiated prophylactically immediately after surgery, or depending on the postoperative parathyroid hormone levels. The prophylactic postoperative supplementation with calcium and vitamin D seems to be more efficient than monotherapy with calcium and can positively affect the development of postoperative hypocalcemia and reduce the need for intravenous calcium supplementation [31]. It was also demonstrated that preoperatively initiated and postoperatively continued calcium and vitamin D supplementation can significantly reduce postoperative hypocalcemia following thyroidectomy [32]. In our study, a similar effect was achieved through preoperative supplementation of calcium and magnesium, as postoperative calcium levels were overall higher in the intervention group. However, there was no significant difference between the groups. Nevertheless, the supplemented GD patients reported fewer symptoms in terms of paresthesia. The extent to which vitamin D deficiency affects quality of life within the groups cannot be determined based on our data. The sample sizes would be too small for a meaningful subgroup analysis. However, given the comparable prevalence of vitamin D deficiency across the groups, we can assume group comparability in this regard.

It has been demonstrated that in thyroidectomised patients who develop postoperative hypocalcemia, serum magnesium levels also significantly decrease within the first two postoperative days. However, this study did not establish a correlation between magnesium levels and the development of hypocalcemia [33]. Minuto et al. also investigated preoperative prophylactic magnesium supplementation but similarly found no correlation between magnesium treatment and the course of postoperative hypocalcemia [34]. In the present study, no association between serum magnesium levels and the development of hypocalcemia could be demonstrated. Furthermore, no hypomagnesemia was observed at any time point in our patient cohort at all. Therefore, the role of magnesium substitution for postoperative hypocalcemia remains unclear, but appears to be of secondary importance.

Vitamin D deficiency is a widespread issue in the German population. Its prevalence is estimated to affect 8.3% of the adult population during the summer months and up to 52% during the winter months [35]. Although the current data is not entirely conclusive, a pre‐existing vitamin D deficiency is generally considered a risk factor for postoperative hypocalcemia. A recent systematic review found a 2.3‐fold increased risk of postoperative hypocalcemia in patients with pre‐existing vitamin D deficiency [36]. However, there are also studies with differing results. For instance, a 2015 study found no association between preoperative vitamin D deficiency and postoperative hypocalcemia in a cohort of 264 patients but they concluded that preoperative Vitamin D status can predict the need for 1,25‐dihydroxyvitamin D3 therapy in hypocalcemic subjects [37]. A multicentre randomised German study, in which participants received calcitriol for 3 days preoperatively, demonstrated that while this did not reduce the rate of hypocalcemia, it did halve the duration of postoperative hypocalcemia [38]. In our study, we did not observe any differences between the groups regarding the impact of pre‐existing vitamin D deficiency on postoperative hypocalcemia. This may be related to the fact that 66.7% of the patient cohort already had a preoperative vitamin D deficiency. A larger patient cohort would be needed to identify potential correlations in this context.

To date, no consensus recommendations or clinical guidelines exist regarding preoperative calcium or magnesium supplementation in patients undergoing thyroidectomy. The rationale for selecting the 2‐week preoperative supplementation period and the chosen dosages was based on the study by Oltmann et al. [39], in which patients with Graves' disease received 3 × 1 g calcium carbonate daily for 2 weeks prior to surgery. To minimise the risk of potential adverse effects, we opted for 3 × 500 mg calcium daily, guided by the EFSA recommendations [40, 41]. Nevertheless, the optimal duration, dosage and potential addition of magnesium supplementation remain topics of ongoing discussion. Whether a shorter preoperative supplementation period might also have a beneficial effect on quality of life warrants further investigation.

## 5 Conclusion

Preoperative supplementation with calcium and magnesium appears to have both a positive effect on the early postoperative improvement in quality of life in patients with Graves' disease and a beneficial influence in preventing postoperative hypocalcemia, even in cases where preoperative vitamin D deficiency is present. In general, a significant improvement in the mean quality of life after thyroidectomy across the entire patient cohort is demonstrated, which seems to be primarily attributable to the surgery itself rather than a supplementation therapy. Oral calcium and magnesium supplementation prior to thyroidectomy, particularly in patients with Graves' disease, might constitute a simple and cost‐effective approach that could help to improve postoperative quality of life and reduce the risk of hypocalcemia.

Further studies, ideally placebo‐controlled, double‐blind and with a larger sample size, are warranted to provide robust general recommendations for preoperative supplementation therapy with calcium (and magnesium) and to evaluate the optimal preoperative duration. An additional assessment at 6 months would be valuable for evaluating long‐term quality of life and for reliably ruling out persistent hypoparathyroidism. The role of additional preoperative magnesium intake remains unclear but appears to be of minor relevance. For future thyroid‐specific studies, the use of a generic questionnaire such as the EQ‐5D may be dispensable when the ThyPRO is employed, as it does not provide additional information.

## Author Contributions

Conceptualization NT; methodology NT; validation: NT, VU, formal analysis: NT, DF, VU, investigation: NT, DF; resources: DW; writing: —original draft preparation NT, DF; writing—review and editing, VU, DW; supervision: NT, DW, project administration; NT, DW.

## Conflicts of Interest

The authors declare no conflicts of interest.

## Data Availability

The data that support the findings of this study are available from the corresponding author upon reasonable request.
